# *catena*-Poly[[[diaqua­(1,10-phenanthroline-κ^2^ *N*,*N*′)zinc]-μ-4-hy­droxy-3-sulfonato­benzoato-κ^2^ *O* ^3^:*O* ^1^] sesquihydrate]

**DOI:** 10.1107/S1600536812018740

**Published:** 2012-05-02

**Authors:** Xiang-Qian Fang, Shan Gao, Seik Weng Ng

**Affiliations:** a Key Laboratory of Functional Inorganic Material Chemistry, Ministry of Education, Heilongjiang University, Harbin 150080, People’s Republic of China; b Department of Chemistry, University of Malaya, 50603 Kuala Lumpur, Malaysia; c Chemistry Department, Faculty of Science, King Abdulaziz University, PO Box 80203 Jeddah, Saudi Arabia

## Abstract

The 1,10-phenanthroline-chelated Zn atom in the polymeric title compound, {[Zn(C_7_H_4_O_6_S)(C_12_H_8_N_2_)(H_2_O)_2_]·1.5H_2_O}_*n*_, is connected to the sulfonate O atom of one 4-hy­droxy-3-sulfonato­benzoate dianion and to the carboxyl­ate O atom of another dianion. It is also coordinated by two water mol­ecules in an overall octa­hedral environment. The dianion links adjacent metal atoms into a chain running along [110]. The chains are linked by O—H⋯O hydrogen bonds into a three-dimensional network.

## Related literature

For the isostructural Mn^II^ derivative, see: Fang *et al.* (2011 ▶) and for the isostructural Co^II^ derivative, see: Fang *et al.* (2012 ▶).
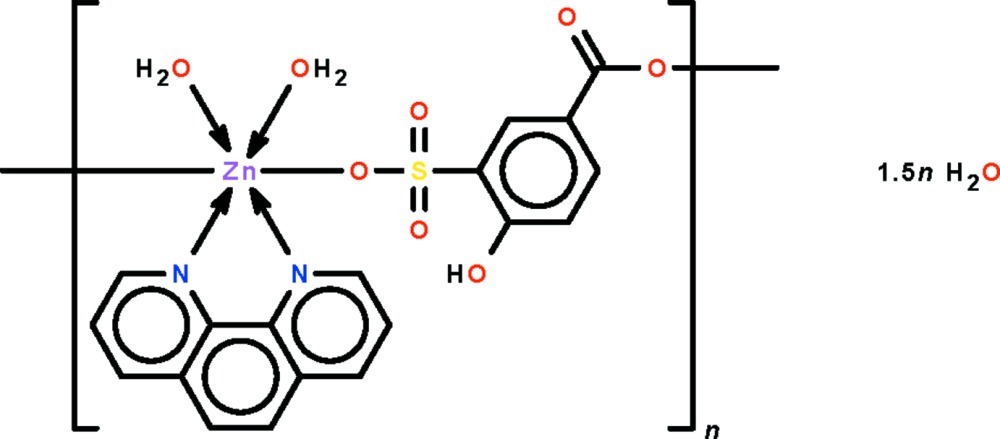


## Experimental

### 

#### Crystal data

[Zn(C_7_H_4_O_6_S)(C_12_H_8_N_2_)(H_2_O)_2_]·1.5H_2_O
*M* *_r_* = 524.79
Monoclinic, 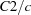

*a* = 8.3682 (3) Å
*b* = 17.3251 (6) Å
*c* = 28.6686 (10) Å
β = 92.848 (1)°
*V* = 4151.2 (3) Å^3^
*Z* = 8
Mo *K*α radiation
μ = 1.34 mm^−1^
*T* = 293 K
0.19 × 0.15 × 0.11 mm

#### Data collection

Rigaku R-AXIS RAPID IP diffractometer
Absorption correction: multi-scan (*ABSCOR*; Higashi, 1995 ▶) *T*_min_ = 0.784, *T*_max_ = 0.866
20011 measured reflections
4741 independent reflections
3098 reflections with *I* > 2σ(*I*)
*R*_int_ = 0.051

#### Refinement

*R*[*F* ^2^ > 2σ(*F* ^2^)] = 0.035
*wR*(*F* ^2^) = 0.104
*S* = 1.16
4741 reflections
326 parameters
8 restraints
H atoms treated by a mixture of independent and constrained refinement
Δρ_max_ = 0.62 e Å^−3^
Δρ_min_ = −0.88 e Å^−3^

### 

Data collection: *RAPID-AUTO* (Rigaku, 1998 ▶); cell refinement: *RAPID-AUTO*; data reduction: *CrystalClear* (Rigaku/MSC, 2002 ▶); program(s) used to solve structure: *SHELXS97* (Sheldrick, 2008 ▶); program(s) used to refine structure: *SHELXL97* (Sheldrick, 2008 ▶); molecular graphics: *X-SEED* (Barbour, 2001 ▶); software used to prepare material for publication: *publCIF* (Westrip, 2010 ▶).

## Supplementary Material

Crystal structure: contains datablock(s) global, I. DOI: 10.1107/S1600536812018740/bt5893sup1.cif

Structure factors: contains datablock(s) I. DOI: 10.1107/S1600536812018740/bt5893Isup2.hkl

Additional supplementary materials:  crystallographic information; 3D view; checkCIF report

## Figures and Tables

**Table 1:** Hydrogen-bond geometry (Å, °)

*D*—H⋯*A*	*D*—H	H⋯*A*	*D*⋯*A*	*D*—H⋯*A*
O4—H4⋯O4w	0.84 (1)	1.78 (1)	2.615 (3)	172 (4)
O1w—H11⋯O6^i^	0.84 (1)	1.68 (1)	2.520 (3)	172 (5)
O1w—H12⋯O3w	0.84 (1)	1.98 (2)	2.793 (2)	164 (4)
O2w—H21⋯O2^ii^	0.84 (1)	1.94 (1)	2.760 (3)	166 (4)
O2w—H22⋯O1w^iii^	0.84 (1)	1.93 (1)	2.767 (3)	174 (4)
O3w—H31⋯O2	0.84 (1)	1.95 (2)	2.752 (3)	160 (4)
O4w—H41⋯O4^iv^	0.84 (1)	2.23 (3)	2.939 (3)	142 (4)
O4w—H42⋯O5^v^	0.84 (1)	1.95 (1)	2.789 (3)	177 (4)

Symmetry codes: (i) 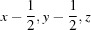; (ii) 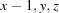; (iii) 
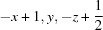
; (iv) 
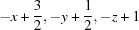
; (v) 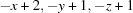.

**Fig. 1.**
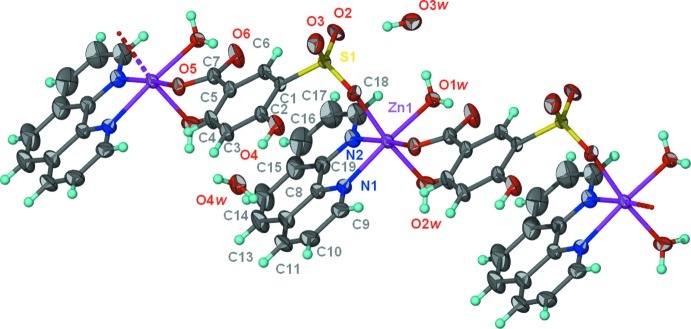
Anisotropic displacement ellipsoid plot (Barbour, 2001) of a portion of the structure of polymeric [Zn(H2O)2(C12H8N2)(C7H4O6S)]n.1.5nH2O at the 50% probability level; hydrogen atoms are drawn as spheres of arbitrary radius.

## supplementary crystallographic information

### Comment

The title zinc compound (Scheme I, Fig. 1) is isostructural with the manganese(II) derivative (Fang *et al.*, 2011) and with the cobalt(II) derivative (Fang *et al.*, 2012). The 1,10-phenanthroline chelated Zn atom is connected to the sulfonate O atom of one (C_7_H_4_O_6_S) dianion and to the carboxylate O atom of another dianion. It is also coordinated by two water molecules in an octahedral environment. The dianion links adjacent metal atoms into a chain along [1 1 0]. The chains are linked by O–H···O hydrogen bonds into a three-dimensional network (Table 1).

### Experimental

A methanol solution (5 ml) of 1,10-phenanthroline (1 mmol) was added to an aqueous solution (10 ml) of zinc(II) dichloride (1 mmol), 2-hydroxy-5-carboxybenzenesulfonic acid (2 mmol) and lithium hydroxide (4 mmol). Colorless crystals were isolated from the solution after several days.

### Refinement

All H atoms were located in a difference map. Carbon-bound H-atoms were placed in calculated positions (C–H 0.93 Å) and were included in the refinement in the riding model approximation, with *U*(H) set to 1.2*U*(C). H-atoms bonded to O were isotropically refined with a distance restraint of O–H 0.84±0.01 Å.

Omitted owing to bad disagreement were (4 0 4), (5 17 4), (-7 11 20), (4 10 27) and (5 11 26).

### Crystal data

**Table:**

[Zn(C_7_H_4_O_6_S)(C_12_H_8_N_2_)(H_2_O)_2_]·1.5H_2_O	*F*(000) = 2152
*M**_r_* = 524.79	*D*_x_ = 1.679 Mg m^−^^3^
Monoclinic, *C*2/*c*	Mo *K*α radiation, λ = 0.71073 Å
Hall symbol: -C 2yc	Cell parameters from 12500 reflections
*a* = 8.3682 (3) Å	θ = 3.0–27.5°
*b* = 17.3251 (6) Å	µ = 1.34 mm^−^^1^
*c* = 28.6686 (10) Å	*T* = 293 K
β = 92.848 (1)°	Prism, colorless
*V* = 4151.2 (3) Å^3^	0.19 × 0.15 × 0.11 mm
*Z* = 8	

### Data collection

**Table:**

Rigaku R-AXIS RAPID IP diffractometer	4741 independent reflections
Radiation source: fine-focus sealed tube	3098 reflections with *I* > 2σ(*I*)
Graphite monochromator	*R*_int_ = 0.051
ω scan	θ_max_ = 27.5°, θ_min_ = 3.0°
Absorption correction: multi-scan (*ABSCOR*; Higashi, 1995)	*h* = −10→10
*T*_min_ = 0.784, *T*_max_ = 0.866	*k* = −18→22
20011 measured reflections	*l* = −37→37

### Refinement

**Table:**

Refinement on *F*^2^	Primary atom site location: structure-invariant direct methods
Least-squares matrix: full	Secondary atom site location: difference Fourier map
*R*[*F*^2^ > 2σ(*F*^2^)] = 0.035	Hydrogen site location: inferred from neighbouring sites
*wR*(*F*^2^) = 0.104	H atoms treated by a mixture of independent and constrained refinement
*S* = 1.16	*w* = 1/[σ^2^(*F*_o_^2^) + (0.0376*P*)^2^ + 5.6207*P*] where *P* = (*F*_o_^2^ + 2*F*_c_^2^)/3
4741 reflections	(Δ/σ)_max_ = 0.001
326 parameters	Δρ_max_ = 0.62 e Å^−^^3^
8 restraints	Δρ_min_ = −0.88 e Å^−^^3^

### Fractional atomic coordinates and isotropic or equivalent isotropic displacement parameters (Å^2^)

**Table:**

	*x*	*y*	*z*	*U*_iso_*/*U*_eq_	
Zn1	0.61591 (4)	0.232574 (19)	0.342240 (11)	0.02942 (12)	
S1	1.01800 (9)	0.26433 (4)	0.37668 (3)	0.02952 (18)	
O1	0.8591 (2)	0.23027 (12)	0.37134 (7)	0.0340 (5)	
O2	1.0907 (3)	0.27321 (13)	0.33170 (8)	0.0489 (7)	
O3	1.1182 (3)	0.22346 (14)	0.41081 (9)	0.0523 (7)	
O4	0.8751 (3)	0.31120 (12)	0.46469 (7)	0.0415 (6)	
O5	1.0863 (3)	0.62949 (11)	0.37689 (7)	0.0331 (5)	
O6	1.1071 (3)	0.54964 (13)	0.31619 (8)	0.0518 (7)	
O1W	0.6923 (3)	0.17550 (13)	0.28105 (7)	0.0314 (5)	
O2W	0.3893 (3)	0.22125 (19)	0.30948 (9)	0.0579 (8)	
O3W	1.0000	0.1982 (2)	0.2500	0.0491 (9)	
O4W	0.7309 (3)	0.35044 (14)	0.53999 (8)	0.0421 (6)	
N1	0.5209 (3)	0.30126 (15)	0.39576 (8)	0.0332 (6)	
N2	0.6531 (3)	0.34633 (15)	0.31609 (9)	0.0374 (6)	
C1	0.9919 (4)	0.35998 (16)	0.39722 (10)	0.0280 (6)	
C2	0.9203 (4)	0.37230 (17)	0.43963 (10)	0.0313 (7)	
C3	0.8988 (4)	0.44787 (18)	0.45453 (11)	0.0385 (8)	
H3	0.8495	0.4571	0.4824	0.046*	
C4	0.9496 (4)	0.50923 (17)	0.42853 (11)	0.0369 (8)	
H4A	0.9348	0.5593	0.4392	0.044*	
C5	1.0230 (4)	0.49733 (17)	0.38647 (10)	0.0318 (7)	
C6	1.0417 (4)	0.42181 (17)	0.37114 (10)	0.0302 (7)	
H6	1.0885	0.4128	0.3429	0.036*	
C7	1.0760 (4)	0.56319 (17)	0.35757 (10)	0.0332 (7)	
C8	0.5305 (4)	0.37811 (19)	0.38722 (11)	0.0377 (8)	
C9	0.4569 (4)	0.2779 (2)	0.43478 (12)	0.0429 (8)	
H9	0.4499	0.2252	0.4406	0.051*	
C10	0.3996 (5)	0.3299 (3)	0.46746 (13)	0.0599 (12)	
H10	0.3567	0.3118	0.4947	0.072*	
C11	0.4075 (5)	0.4066 (3)	0.45891 (15)	0.0636 (12)	
H11A	0.3691	0.4414	0.4803	0.076*	
C12	0.4733 (5)	0.4339 (2)	0.41789 (14)	0.0517 (10)	
C13	0.4894 (6)	0.5136 (2)	0.40649 (19)	0.0738 (14)	
H13	0.4525	0.5509	0.4267	0.089*	
C14	0.5563 (6)	0.5357 (2)	0.3673 (2)	0.0795 (15)	
H14	0.5647	0.5881	0.3609	0.095*	
C15	0.6156 (5)	0.4811 (2)	0.33509 (15)	0.0564 (11)	
C16	0.6890 (6)	0.5010 (2)	0.29391 (17)	0.0740 (14)	
H16	0.7015	0.5528	0.2862	0.089*	
C17	0.7418 (6)	0.4454 (3)	0.26522 (15)	0.0701 (13)	
H17	0.7913	0.4584	0.2380	0.084*	
C18	0.7207 (5)	0.3679 (2)	0.27727 (12)	0.0506 (10)	
H18	0.7555	0.3300	0.2572	0.061*	
C19	0.6008 (4)	0.40212 (18)	0.34510 (11)	0.0376 (8)	
H4	0.832 (5)	0.328 (2)	0.4885 (9)	0.066 (13)*	
H11	0.665 (6)	0.1314 (13)	0.2903 (16)	0.092 (17)*	
H12	0.7903 (16)	0.176 (2)	0.2762 (13)	0.054 (12)*	
H21	0.301 (3)	0.232 (2)	0.3201 (14)	0.060 (12)*	
H22	0.368 (5)	0.204 (2)	0.2826 (7)	0.066 (13)*	
H31	1.023 (5)	0.2307 (17)	0.2710 (10)	0.055 (12)*	
H41	0.669 (4)	0.3148 (17)	0.5466 (14)	0.064 (13)*	
H42	0.784 (4)	0.358 (2)	0.5651 (8)	0.060 (13)*	

### Atomic displacement parameters (Å^2^)

**Table:**

	*U*^11^	*U*^22^	*U*^33^	*U*^12^	*U*^13^	*U*^23^
Zn1	0.0375 (2)	0.02202 (19)	0.02921 (19)	−0.00039 (15)	0.00628 (15)	−0.00082 (14)
S1	0.0306 (4)	0.0221 (4)	0.0363 (4)	−0.0016 (3)	0.0063 (3)	−0.0058 (3)
O1	0.0303 (11)	0.0265 (11)	0.0448 (12)	−0.0081 (9)	−0.0009 (10)	−0.0059 (9)
O2	0.0613 (16)	0.0375 (14)	0.0506 (14)	−0.0070 (12)	0.0310 (13)	−0.0130 (11)
O3	0.0504 (16)	0.0372 (14)	0.0673 (17)	0.0087 (12)	−0.0172 (13)	−0.0003 (12)
O4	0.0697 (17)	0.0242 (11)	0.0324 (12)	−0.0106 (11)	0.0196 (12)	−0.0017 (9)
O5	0.0482 (14)	0.0223 (11)	0.0292 (10)	−0.0077 (10)	0.0066 (10)	−0.0020 (9)
O6	0.094 (2)	0.0289 (13)	0.0343 (12)	−0.0179 (13)	0.0245 (13)	−0.0051 (10)
O1W	0.0369 (13)	0.0288 (12)	0.0295 (11)	−0.0043 (10)	0.0108 (10)	−0.0007 (9)
O2W	0.0366 (15)	0.100 (2)	0.0372 (14)	0.0095 (15)	0.0010 (13)	−0.0240 (15)
O3W	0.047 (2)	0.055 (2)	0.045 (2)	0.000	0.0070 (19)	0.000
O4W	0.0600 (17)	0.0377 (14)	0.0292 (12)	−0.0082 (12)	0.0086 (12)	−0.0005 (10)
N1	0.0329 (14)	0.0366 (15)	0.0302 (13)	0.0011 (12)	0.0038 (12)	−0.0056 (11)
N2	0.0476 (17)	0.0278 (14)	0.0372 (14)	0.0006 (12)	0.0052 (13)	0.0011 (11)
C1	0.0295 (16)	0.0228 (15)	0.0319 (15)	−0.0045 (12)	0.0043 (13)	−0.0046 (12)
C2	0.0408 (18)	0.0253 (15)	0.0283 (15)	−0.0090 (13)	0.0061 (14)	−0.0004 (12)
C3	0.058 (2)	0.0294 (17)	0.0296 (16)	−0.0062 (15)	0.0174 (16)	−0.0054 (13)
C4	0.055 (2)	0.0228 (16)	0.0336 (16)	−0.0083 (15)	0.0118 (16)	−0.0082 (13)
C5	0.0423 (19)	0.0231 (15)	0.0305 (15)	−0.0064 (13)	0.0067 (14)	−0.0005 (12)
C6	0.0371 (17)	0.0295 (16)	0.0249 (14)	−0.0059 (13)	0.0094 (13)	−0.0027 (12)
C7	0.0427 (19)	0.0251 (16)	0.0323 (16)	−0.0073 (14)	0.0069 (15)	−0.0047 (13)
C8	0.0375 (18)	0.0310 (17)	0.0438 (18)	0.0069 (14)	−0.0054 (15)	−0.0119 (15)
C9	0.0344 (18)	0.056 (2)	0.0386 (18)	0.0004 (16)	0.0027 (15)	−0.0060 (16)
C10	0.046 (2)	0.091 (4)	0.044 (2)	0.000 (2)	0.0122 (19)	−0.019 (2)
C11	0.052 (3)	0.079 (3)	0.060 (3)	0.013 (2)	0.004 (2)	−0.038 (2)
C12	0.046 (2)	0.050 (2)	0.059 (2)	0.0124 (18)	−0.0039 (19)	−0.0237 (19)
C13	0.081 (3)	0.044 (3)	0.095 (4)	0.023 (2)	−0.004 (3)	−0.034 (3)
C14	0.106 (4)	0.025 (2)	0.107 (4)	0.009 (2)	−0.004 (3)	−0.014 (2)
C15	0.068 (3)	0.0264 (18)	0.074 (3)	0.0044 (18)	−0.007 (2)	0.0001 (18)
C16	0.105 (4)	0.032 (2)	0.085 (3)	−0.009 (2)	0.002 (3)	0.019 (2)
C17	0.099 (4)	0.050 (3)	0.061 (3)	−0.013 (3)	0.011 (3)	0.020 (2)
C18	0.070 (3)	0.037 (2)	0.045 (2)	−0.0027 (18)	0.013 (2)	0.0070 (16)
C19	0.042 (2)	0.0239 (16)	0.0462 (19)	0.0049 (14)	−0.0069 (16)	−0.0018 (14)

### Geometric parameters (Å, º)

**Table:**

Zn1—O5^i^	2.065 (2)	C2—C3	1.392 (4)
Zn1—O2W	2.083 (3)	C3—C4	1.378 (4)
Zn1—N1	2.127 (2)	C3—H3	0.9300
Zn1—N2	2.137 (3)	C4—C5	1.395 (4)
Zn1—O1W	2.139 (2)	C4—H4A	0.9300
Zn1—O1	2.161 (2)	C5—C6	1.392 (4)
S1—O3	1.442 (3)	C5—C7	1.491 (4)
S1—O1	1.456 (2)	C6—H6	0.9300
S1—O2	1.461 (2)	C8—C12	1.407 (4)
S1—C1	1.776 (3)	C8—C19	1.431 (5)
O4—C2	1.344 (3)	C9—C10	1.401 (5)
O4—H4	0.839 (10)	C9—H9	0.9300
O5—C7	1.276 (3)	C10—C11	1.354 (6)
O5—Zn1^ii^	2.065 (2)	C10—H10	0.9300
O6—C7	1.249 (4)	C11—C12	1.405 (6)
O1W—H11	0.844 (10)	C11—H11A	0.9300
O1W—H12	0.839 (10)	C12—C13	1.427 (6)
O2W—H21	0.835 (10)	C13—C14	1.335 (6)
O2W—H22	0.836 (10)	C13—H13	0.9300
O3W—H31	0.839 (10)	C14—C15	1.429 (6)
O4W—H41	0.836 (10)	C14—H14	0.9300
O4W—H42	0.838 (10)	C15—C16	1.401 (6)
N1—C9	1.327 (4)	C15—C19	1.404 (5)
N1—C8	1.357 (4)	C16—C17	1.356 (6)
N2—C18	1.327 (4)	C16—H16	0.9300
N2—C19	1.362 (4)	C17—C18	1.398 (5)
C1—C6	1.383 (4)	C17—H17	0.9300
C1—C2	1.398 (4)	C18—H18	0.9300
			
O5^i^—Zn1—O2W	90.42 (11)	C3—C4—H4A	119.5
O5^i^—Zn1—N1	94.52 (9)	C5—C4—H4A	119.5
O2W—Zn1—N1	90.76 (10)	C6—C5—C4	118.2 (3)
O5^i^—Zn1—N2	171.77 (9)	C6—C5—C7	120.2 (3)
O2W—Zn1—N2	94.24 (12)	C4—C5—C7	121.6 (3)
N1—Zn1—N2	78.66 (10)	C1—C6—C5	121.0 (3)
O5^i^—Zn1—O1W	92.44 (8)	C1—C6—H6	119.5
O2W—Zn1—O1W	83.65 (9)	C5—C6—H6	119.5
N1—Zn1—O1W	171.10 (10)	O6—C7—O5	124.7 (3)
N2—Zn1—O1W	94.81 (9)	O6—C7—C5	117.8 (3)
O5^i^—Zn1—O1	86.06 (8)	O5—C7—C5	117.6 (3)
O2W—Zn1—O1	172.29 (10)	N1—C8—C12	122.4 (3)
N1—Zn1—O1	96.35 (9)	N1—C8—C19	117.9 (3)
N2—Zn1—O1	90.09 (10)	C12—C8—C19	119.6 (3)
O1W—Zn1—O1	89.65 (8)	N1—C9—C10	122.3 (4)
O3—S1—O1	111.69 (15)	N1—C9—H9	118.9
O3—S1—O2	113.39 (16)	C10—C9—H9	118.9
O1—S1—O2	111.59 (14)	C11—C10—C9	119.2 (4)
O3—S1—C1	108.07 (14)	C11—C10—H10	120.4
O1—S1—C1	106.63 (13)	C9—C10—H10	120.4
O2—S1—C1	104.94 (13)	C10—C11—C12	120.5 (3)
S1—O1—Zn1	150.55 (14)	C10—C11—H11A	119.8
C2—O4—H4	108 (3)	C12—C11—H11A	119.8
C7—O5—Zn1^ii^	125.22 (19)	C11—C12—C8	116.8 (4)
Zn1—O1W—H11	94 (3)	C11—C12—C13	124.3 (4)
Zn1—O1W—H12	118 (3)	C8—C12—C13	118.9 (4)
H11—O1W—H12	111 (4)	C14—C13—C12	121.2 (4)
Zn1—O2W—H21	128 (3)	C14—C13—H13	119.4
Zn1—O2W—H22	127 (3)	C12—C13—H13	119.4
H21—O2W—H22	105 (4)	C13—C14—C15	121.8 (4)
H41—O4W—H42	103 (4)	C13—C14—H14	119.1
C9—N1—C8	118.8 (3)	C15—C14—H14	119.1
C9—N1—Zn1	128.2 (2)	C16—C15—C19	117.4 (4)
C8—N1—Zn1	113.01 (19)	C16—C15—C14	124.2 (4)
C18—N2—C19	118.4 (3)	C19—C15—C14	118.5 (4)
C18—N2—Zn1	129.1 (2)	C17—C16—C15	120.3 (4)
C19—N2—Zn1	112.6 (2)	C17—C16—H16	119.8
C6—C1—C2	120.4 (3)	C15—C16—H16	119.8
C6—C1—S1	119.9 (2)	C16—C17—C18	118.9 (4)
C2—C1—S1	119.7 (2)	C16—C17—H17	120.5
O4—C2—C3	122.2 (3)	C18—C17—H17	120.5
O4—C2—C1	119.3 (3)	N2—C18—C17	122.8 (4)
C3—C2—C1	118.6 (3)	N2—C18—H18	118.6
C4—C3—C2	120.8 (3)	C17—C18—H18	118.6
C4—C3—H3	119.6	N2—C19—C15	122.2 (3)
C2—C3—H3	119.6	N2—C19—C8	117.9 (3)
C3—C4—C5	121.0 (3)	C15—C19—C8	120.0 (3)
			
O3—S1—O1—Zn1	173.2 (2)	Zn1^ii^—O5—C7—C5	−168.2 (2)
O2—S1—O1—Zn1	−58.7 (3)	C6—C5—C7—O6	13.8 (5)
C1—S1—O1—Zn1	55.4 (3)	C4—C5—C7—O6	−164.3 (3)
O5^i^—Zn1—O1—S1	179.0 (3)	C6—C5—C7—O5	−166.3 (3)
N1—Zn1—O1—S1	−86.9 (3)	C4—C5—C7—O5	15.7 (5)
N2—Zn1—O1—S1	−8.3 (3)	C9—N1—C8—C12	−0.9 (5)
O1W—Zn1—O1—S1	86.5 (3)	Zn1—N1—C8—C12	179.4 (3)
O5^i^—Zn1—N1—C9	−4.3 (3)	C9—N1—C8—C19	179.6 (3)
O2W—Zn1—N1—C9	86.1 (3)	Zn1—N1—C8—C19	−0.1 (4)
N2—Zn1—N1—C9	−179.7 (3)	C8—N1—C9—C10	−0.2 (5)
O1—Zn1—N1—C9	−90.9 (3)	Zn1—N1—C9—C10	179.5 (3)
O5^i^—Zn1—N1—C8	175.4 (2)	N1—C9—C10—C11	0.8 (6)
O2W—Zn1—N1—C8	−94.2 (2)	C9—C10—C11—C12	−0.4 (6)
N2—Zn1—N1—C8	0.0 (2)	C10—C11—C12—C8	−0.6 (6)
O1—Zn1—N1—C8	88.8 (2)	C10—C11—C12—C13	−178.7 (4)
O2W—Zn1—N2—C18	−91.4 (3)	N1—C8—C12—C11	1.2 (5)
N1—Zn1—N2—C18	178.7 (3)	C19—C8—C12—C11	−179.2 (3)
O1W—Zn1—N2—C18	−7.4 (3)	N1—C8—C12—C13	179.5 (4)
O1—Zn1—N2—C18	82.3 (3)	C19—C8—C12—C13	−1.0 (5)
O2W—Zn1—N2—C19	90.1 (2)	C11—C12—C13—C14	178.5 (5)
N1—Zn1—N2—C19	0.1 (2)	C8—C12—C13—C14	0.4 (7)
O1W—Zn1—N2—C19	174.0 (2)	C12—C13—C14—C15	−0.2 (8)
O1—Zn1—N2—C19	−96.3 (2)	C13—C14—C15—C16	−179.0 (5)
O3—S1—C1—C6	120.0 (3)	C13—C14—C15—C19	0.6 (7)
O1—S1—C1—C6	−119.8 (3)	C19—C15—C16—C17	0.4 (7)
O2—S1—C1—C6	−1.3 (3)	C14—C15—C16—C17	180.0 (5)
O3—S1—C1—C2	−60.3 (3)	C15—C16—C17—C18	0.5 (8)
O1—S1—C1—C2	59.9 (3)	C19—N2—C18—C17	0.7 (6)
O2—S1—C1—C2	178.4 (3)	Zn1—N2—C18—C17	−177.8 (3)
C6—C1—C2—O4	−179.0 (3)	C16—C17—C18—N2	−1.1 (7)
S1—C1—C2—O4	1.3 (4)	C18—N2—C19—C15	0.3 (5)
C6—C1—C2—C3	0.8 (5)	Zn1—N2—C19—C15	179.1 (3)
S1—C1—C2—C3	−178.9 (3)	C18—N2—C19—C8	−179.0 (3)
O4—C2—C3—C4	178.6 (3)	Zn1—N2—C19—C8	−0.2 (4)
C1—C2—C3—C4	−1.3 (5)	C16—C15—C19—N2	−0.8 (6)
C2—C3—C4—C5	0.4 (6)	C14—C15—C19—N2	179.5 (4)
C3—C4—C5—C6	0.8 (5)	C16—C15—C19—C8	178.5 (4)
C3—C4—C5—C7	178.9 (3)	C14—C15—C19—C8	−1.2 (6)
C2—C1—C6—C5	0.4 (5)	N1—C8—C19—N2	0.3 (5)
S1—C1—C6—C5	−179.8 (3)	C12—C8—C19—N2	−179.3 (3)
C4—C5—C6—C1	−1.2 (5)	N1—C8—C19—C15	−179.1 (3)
C7—C5—C6—C1	−179.4 (3)	C12—C8—C19—C15	1.4 (5)
Zn1^ii^—O5—C7—O6	11.8 (5)		

Symmetry codes: (i) *x*−1/2, *y*−1/2, *z*; (ii) *x*+1/2, *y*+1/2, *z*.

### Hydrogen-bond geometry (Å, º)

**Table:**

*D*—H···*A*	*D*—H	H···*A*	*D*···*A*	*D*—H···*A*
O4—H4···O4w	0.84 (1)	1.78 (1)	2.615 (3)	172 (4)
O1w—H11···O6^i^	0.84 (1)	1.68 (1)	2.520 (3)	172 (5)
O1w—H12···O3w	0.84 (1)	1.98 (2)	2.793 (2)	164 (4)
O2w—H21···O2^iii^	0.84 (1)	1.94 (1)	2.760 (3)	166 (4)
O2w—H22···O1w^iv^	0.84 (1)	1.93 (1)	2.767 (3)	174 (4)
O3w—H31···O2	0.84 (1)	1.95 (2)	2.752 (3)	160 (4)
O4w—H41···O4^v^	0.84 (1)	2.23 (3)	2.939 (3)	142 (4)
O4w—H42···O5^vi^	0.84 (1)	1.95 (1)	2.789 (3)	177 (4)

Symmetry codes: (i) *x*−1/2, *y*−1/2, *z*; (iii) *x*−1, *y*, *z*; (iv) −*x*+1, *y*, −*z*+1/2; (v) −*x*+3/2, −*y*+1/2, −*z*+1; (vi) −*x*+2, −*y*+1, −*z*+1.
